# The effect of platelet lysate in culture of PDLSCs: an *in vitro* comparative study

**DOI:** 10.7717/peerj.7465

**Published:** 2019-08-08

**Authors:** Duaa A. Abuarqoub, Nazneen Aslam, Raghda B. Barham, Nidaa A. Ababneh, Diana A. Shahin, Abdallah A. Al-oweidi, Hanan D. Jafar, Mazin A. Al-Salihi, Abdalla S. Awidi

**Affiliations:** 1 Cell Therapy Center, The University of Jordan, Amman, Jordan; 2 School of Medicine, The University of Jordan, Amman, Jordan

**Keywords:** Enzymatic digestion, PL, Explant, FBS, PDLSCs, Osteogenic differentation

## Abstract

**Background:**

Cellular therapy clinical applications require large-scale production of stem cells. Therefore, abundance, ease of isolation, and proliferative potential are the most important factors in choosing the appropriate source of cells for transplantation studies. Multipotent stem cells obtained from periodontal ligament (PDL) can be used in periodontal tissue regeneration. In this study, we aimed to evaluate and compare the characteristics of periodontal ligament stem cells (PDLSCs), extracted by either enzymatic digestion or explant methods, and expanded using two different serum types: fetal bovine serum (FBS) and xeno-free platelet lysate (PL).

**Methods:**

Expanded PDLSCs were assessed for their proliferation capacity, surface markers expression, colony formation, differentiation potential and ability to self-renewal. Most importantly, PDLSCs were evaluated for their ability to produce osteoblasts *in vitro*.

**Results:**

PDLSCs isolated by explant method and expanded in PL serve as a promising source of stem cells for osteoblasts regeneration. These cells showed higher proliferation capacity, they retained their stemness characteristics throughout the passages and they revealed an increase in the expression level of osteogenic markers, without showing any karyotypic abnormalities after cell expansion.

**Conclusions:**

PDLSCs produced using explant extraction method and expanded in cell culture media supplemented with PL provide an excellent source of xeno-free cells for the generation of functional osteoblasts.

## Introduction

Periodontitis is characterized by the destruction of the periodontium, including gingiva, periodontal ligament (PDL), cementum, and alveolar bone (Kiran et al., 2005). Without adequate treatment, periodontitis eventually leads to tooth loss (Albandar, 2005; Baelum & Lopez, 2013). Current treatments are generally successful in preventing active disease, but the regeneration of lost tissues remains a challenge. Thus, research is oriented toward finding new alternative treatments that can compensate the lost tissues. Donated teeth derived stem cells-based approaches provide a novel therapeutic paradigm for periodontal regeneration (Liu et al., 2012; Washio et al., 2010).

Dental stem cells derived from the periodontal ligament (PDLSCs) can be used in periodontal regeneration after differentiating them into cementoblasts (Seo et al., 2004) and osteoblasts (Moshaverinia et al., 2014). However, primary cultures of PDLSCs typically yield small number of cells, which is not enough to generate cell sheets to be used in the regeneration of lost tissues (Feng et al., 2010). Therefore, expanding PDLSCs *in vitro* without losing their stemness properties is of paramount importance for any future transplantation strategies (Laino et al., 2005; Zhu & Liang, 2015).

Most of the previously published PDLSCs isolation and expansion protocols used culture media supplemented with bovine serum proteins for clinical-scale production of stem cells (Gombold et al., 2014; Healy et al., 2005; Tarle, Shi & Kaigler, 2011; Washio et al., 2010). These proteins can potentially stimulate immunogenicity (Chachques et al., 2004; Martin et al., 2005; Spees et al., 2004) and introduce viral and prions contamination (WHO, 1997; Halme & Kessler, 2006). On the other hand, xeno-free culture medium has been used as an efficient alternative to fetal bovine serum (FBS) in culturing several types of dental stem cells, with lower risk of immunogenicity or contamination and higher capacity to maintain genomic stability and multipotency (Abuarqoub, Awidi & Abuharfeil, 2015; Trubiani et al., 2015). In other studies, the use of autologous serum has been recommended for the cryopreservation of cells instead of using any animal-derived serum (Mealey & Rose, 2008; Osathanon, 2010; Zhu & Liang, 2015).

Nowadays, the application of good manufacturing practice (GMP) is required for manufacturing cellular products for any intended therapeutic use. Recently, platelet lysate (PL) has drawn a lot of attention as an alternative to FBS for the preparation of clinical-grade cell expansion (Schallmoser et al., 2007). Platelet granules are rich in growth factors such as: platelet derived growth factor (PDGF), fibroblast growth factor (FGF), insulin growth factor (IGF), and transforming growth factor-b (TGFB) (Schallmoser et al., 2007; Schallmoser & Strunk, 2013). A number of previously published studies have reported the effectiveness of using PL in clinical trials for both autologous and allogeneic transplantations (Al-Ajlouni et al., 2015; Espinosa et al., 2015; Kojima et al., 2008).

Few studies have investigated the immunophenotypic, morphologic and proliferative patterns of PDL cells. (Feng et al., 2010; Iwata et al., 2010). Multipotent stem cells isolated from PDL, either by enzymatic dissociation or explant methods, can be expanded *in vitro* to produce high number of cells for cryopreservation (Tanaka et al., 2011; Tran Hle et al., 2014). However, their characterization using different types of serum still needs to be further investigated. Previous studies have compared the explant and enzymatic digestion approaches on producing mesenchymal stem cells (MSCs) from umbilical cord samples (Salehinejad et al., 2012; Sobolewski et al., 2005). These studies showed that the explant extraction method has many advantages compared to enzymatic digestion. First, the explant extraction method produced high yield of cells with low cost compared to the enzymatic protocols (Han et al., 2013). Second, explant derived cells showed shorter doubling time with increased cellular viability (Han et al., 2013; Sobolewski et al., 2005). Third, many growth factors can be released from tissue pieces during the *in vitro* culture of the explant derived cells to regulate their self-renewal ability and enhance their multilineage differentiation potential (Han et al., 2013; Sobolewski et al., 2005). Finally, explants method reduces the possibility of cellular damage during sample preparation, which could easily be induced by enzymatic digestion (Tsutsumi et al., 2001).

In this study, we aimed to compare the cellular characteristics of PDLSCs extracted by the aforementioned protocols and expanded in two different serum types, fetal bovine serum (FBS) and platelet lysate (PL), to demonstrate the multipotency characteristics of the isolated cells for any future transplantation strategies.

## Material and Methods

### Sample collection

After study description, signed informed consents (Form- S1) were obtained from all donors before inclusion in this study. Third molar teeth were collected from three donors aged 18, 19 and 21 years. All donors showed no clinically evident disease or history of medications, smoking or alcohol consumption.

### Platelet lysate preparation

Sample collection was performed according to the Institutional Review Board (IRB) guidelines from the Cell Therapy Center/University of Jordan (99/2014/TRBJ). Blood samples were collected in Citrate tubes (BD Vacutainer, USA) and centrifuged at 192 xg for 13 min at 4 °C (3-16KL; Sigma, St. Louis, MO, USA). After centrifugation, supernatants were pooled from multiple donor samples and frozen at −80 °C until further use. To prepare platelet rich plasma (PRP), pooled samples were centrifuged at 192 xg for 10 min and followed by two rapidfreeze-thaw cycles (−80 °C and 37 °C). The resulting human PLwas centrifuged for 10 min at 3,894 xg and 4 °C to remove the platelet bodies. And finally, the PL was filtered through 0.22 µm filters (TPP, USA) to remove the platelet membranes (Abuarqoub, Awidi & Abuharfeil, 2015). 

### PDLSCs isolation

PDL tissues were either undergone enzymatic dissociation or explants culture for the isolation of PDLSCs.

1. *Enzymatic dissociation method*

For enzymatic digestion, first the teeth were disinfected with PBS containing 10% Pencillin / Streptomycin (Gibco, Waltham, MA, USA). Then, PDL tissue was dissected from the mid third level of the root and digested in a solution containing 3 mg/mlcollagenase type I (GIBCO, Waltham, MA, USA) and 4 mg/ml dispase (GIBCO, Waltham, MA, USA) for 1 h at 37 °C. Single-cell suspensions were obtained by passing the cells through 70 µm cell strainer (BD, USA) and then cells were centrifuged at 1,000 rpm for 5 min. The resulting pellet was re-suspended in 1 ml of cell culture media, seeded at 5,000 cells/cm^2^ seeding density in 6-well plates and maintained in alpha-Modification ofEagle’s Medium(*α*-MEM, GIBCO, USA) supplemented with 100 µg/ml penicillin/streptomycin (Invitrogen, Waltham, MA, USA), 2 mM L-glutamine (Invitrogen, USA), 0.25 µg/ml Amphotericin B (Invitrogen, Waltham, MA, USA) and 5% PL. The cells were incubated at 37 °C in 5% CO_2_, and medium was exchanged every three days until the cells reached the confluency. The morphological appearance was observed under the inverted microscope (Zeiss, Cincinatti, OH, USA), images were taken on day 7 of the primary culture. 

2. *Explant method*

Each PDL tissue sample was cut into 1–2-mm fragments, and individual pieces were placed in a 6-well culture plate with a minimal amount of *α*-MEM medium supplemented with PL. Plates were kept in the incubator at 37 °C and 5% CO_2_ till the attachment of tissue fragments. Medium was exchanged every 3 days until the outgrowth of the PDLSCs was observed. The morphological appearance was observed under the inverted microscope (Zeiss, USA), images were taken on day 7 of the primary culture. 

### Immunophenotyping using flow cytometry

To assess the expression of MSC surface markers, PDLSCs were harvested at passage 3 and washed twice with PBS. Approximately, 2 × 10^6^ cells were incubated for half an hour at room temperature (RT) with fluorescin-labeled antibodies using the mesenchymal stem cell characterization kit (stem flow kit; BD Biosciences, Franklin Lakes, NJ, USA). The kit contains antibodies for the following MSC markers: PerCP Cy5.5-CD105, FITC-CD90, PE-CD44 and APC-CD73 and the PE-Negative cocktail hematopoietic stem cell (HSC) markers (CD34, CD45, Cd11b and HLA-DR) and their isotype controls. After incubation, the cells were centrifuged at 300 xg for 5 min andthen re-suspended in PBS. Antibodyconcentration used was based on the manufacturer’s instructions (BD, Franklin Lakes, NJ, USA). The expression profile was analyzed by Flourescience Activated Cell Sorter FACS Canto II (BD, Franklin Lakes, NJ, USA). 

### Cell proliferation assay

 A total of 2500 PDLSCs at passage 3 (P3) isolated either by enzymatic or tissue explant protocols were seeded into a 96-well plate (TPP, Trasadingen, Switzerland) and cultured in media containing either 5% PL, 10% FBS or serum-free medium (SFM). The experiment was performed daily for a total of 7 days and media were exchanged every 3 days. Proliferation rate was measured using CellTiter 96® Non-Radioactive Cell Proliferation Assay (Promega, Madison, WI, USA).

### Colony formation unit assay (CFU)

A total of 500 PDLSCs at P1 through 5 were obtained from enzymatic and explant methods and seeded into 20-mm cell culture dishes in either 10% FBS, 5% PL or SFM. Cells were cultured for 14 days with medium exchange every 3 days. Aggregates consisted of more than 50 cells were recognized as PDLSCs colonies. Colonies were stained using Crystal Violet dye (Sigma, St. Louis, MO, USA) followed by colonies counting under the inverted microscope (Zeiss, Cincinatti, OH, USA). 

### Karyotyping

Cytogenic analysis was performed at passage 3, 5 and 7 for PDLSCs isolated by either enzymatic or explants and propagated in two different serum types. Briefly at 70% confluency, cells were treated with Colcemid 10 µg/mL (Euroclone, Italy) for a period of 4–6 h to induce cell cycle arrest at metaphase. Cells were trypsinized and centrifuged at 400 xg for 5 min. The cell pellet was resuspended in a volume less than or equal to 500 ml of alpha-MEM (Gibco, Waltham, MA, USA) and then subjected to 0.075 M KCl hypotonic solution (Euroclone, Pero, Italy), added drop by drop under continuous agitation. After 30 min of incubation at 37 °C and in the presence of KCl, cells were centrifuged at 400 xg for 10 min. Next, The cell pellet was fixed with 10 ml freshly prepared Carnoy’s solution consisting 3;1 methanol:acetic acid (Carlo Erba Regeants) for 20 min. Then, the pellet was washed twice with the same solution. After the final washing step, the pellet was resuspended in 3 ml of the same solution (carnoy’s solution), and dropped on pre-cooled glass slides. Slides were then stained using the classical GTG (G-bands after trypsin and giemsa) and the number of cells in metaphases were analysed under the light microscope at 100 X magnification and using oil immersion. A minimum of 7 metaphases were counted per sample. The analysis of these cells was performed using an imager D2 microscope (Zeiss) and images were captured using the Karyo Vision 3.1 imaging software (Borgonovo et al., 2014).

### Stemness assay

Cells were passaged until P10 in either 10%  FBS or 5% PL to evaluate the self-renewal capabilities of PDLSCs. Then cells were trypsinized after reaching 80% confluency, stained with trypan blue (Gibco) and counted using a haemocytometer. For each passage, the seeding density was 10^5^ cells/25 cm^2^ tissue culture flask. Stemness characteristics were also assessed by flow cytometry for the expression of the following stem cell markers: CD90, CD44, CD73, CD105 and the negative cocktail markers (CD34, CD45, Cd11b and HLA-DR).

### Osteogenic Differentiation

For osteogenic differentiation, PDLSCs at P3 from enzymatic and explant methods were plated at 1 × 10^5^ cells per well in 6-well plates, and maintained in growth media containing either 10% FBS or 5% PL until they reached 70% confluence. After that, growth medium was replaced withosteogenic media and maintained under the same serum conditions. Osteogenic media was prepared using α-MEM supplemented with 10 mM  β-glycerophosphate (SigmaAldrich), 50 µg/ml L-ascorbic acid 2-phosphate (Sigma-Aldrich) and 1 µM dexamethasone (Sigma-Aldrich). Medium was exchanged every 3 days for a total of 14 days after which they were processed for RNA isolation and Alizarin Red staining. 

The functionality of the cultured osteoblasts was examined by quantitative PCR (qPCR) for the following osteogenic markers: osteocalcin (OCN), alkaline phosphatase (ALP), collagen type 1, osteopontin (OPN), Osterix and Cbfa-1. Briefly, RNA was extracted from the differentiated samples, and then cDNA synthesis was performed using superscript VILO master mix (Thermo Fisher). Diluted cDNA was amplified using GoTaq® qPCR Master mix (Promega) and mixed with 300 nM of each of the forward and reverse primers (IDT, USA) (Table S1). The amplification conditions were as follow: 95 °C for 2 min as initial denaturation cycle, then 40 cycles of 95 °C for 15 s, 60 s of annealing step at 58 °C for each of OCN, OPN and ALP genes and 60 °C for each of Osterix, collagen type 1 and Cbfa-1, and 72 °C for 30 s as an extension step. Samples were run on CFX system (Bio-Rad, Hercules, CA, USA) and the relative expression levels of these genes were normalized to cDNA samples from control cells harvested at day 14 (cells cultured in osteogenic supplements-free media under the same conditions). Moreover, the relative expression levels of osteogenic genes were also normalized to cDNA samples of cells cultured under the same conditions and harvested at day1 of the differentiation process.

### Statistical analysis

Data were analyzed on GraphPad Prism version 7. All assays were performed in duplicate of at least three independent experiments (*n* = 3). Results are expressed as Mean ± Standard Deviation. Statistical significance was determined using either a paired *t*-test or one-way analysis of variance (Heider et al., 2013) as appropriate. Statistical significant difference was considered when *p*-value < 0.05.

## Results

### Morphological evaluation of PDLSCs

**Figure 1:**
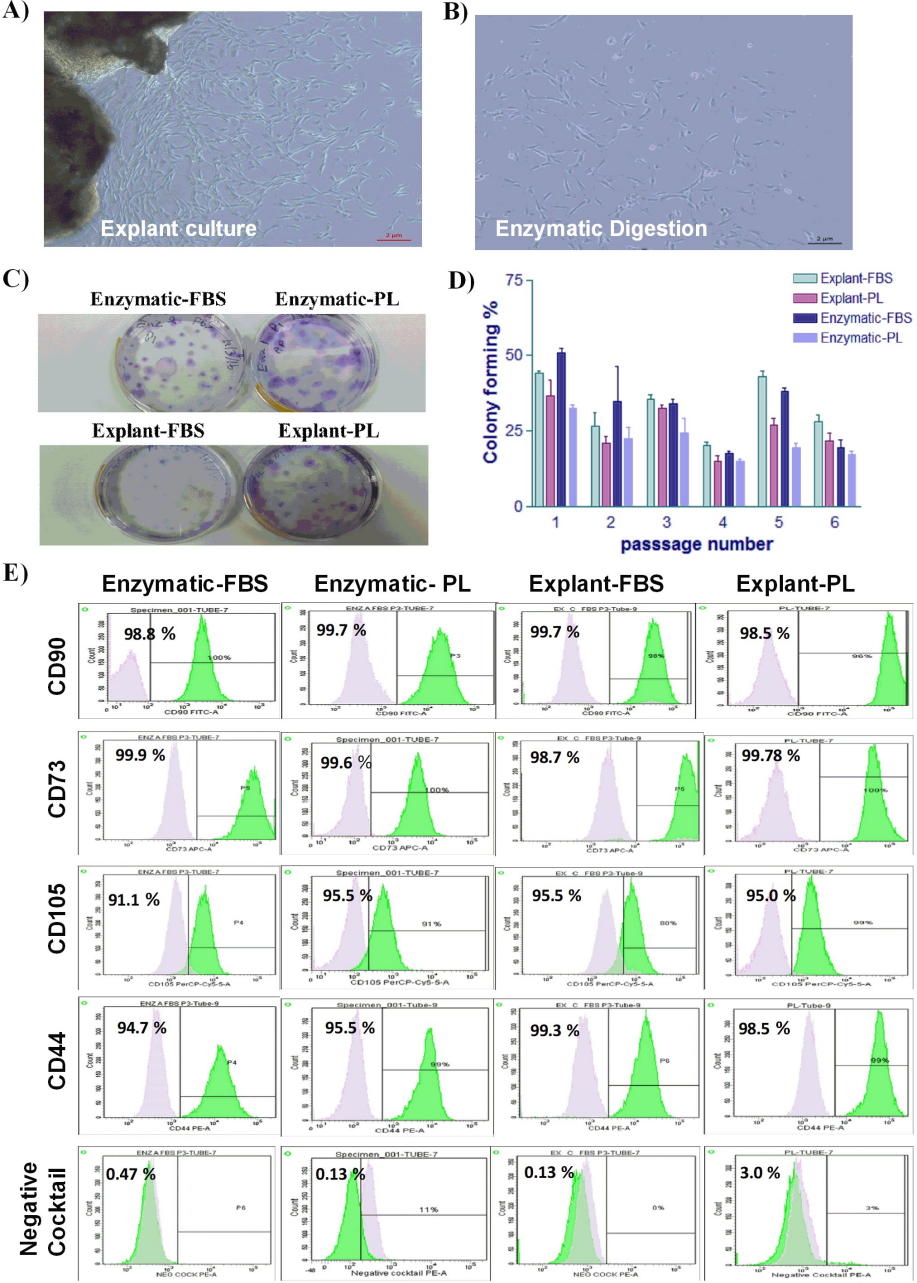
Characterization of PDLSCs. (A & B) The morphological appearance of P0 PDLSCs at day 7, isolated by either explant or enzymatic method. Both cell types showed similarmorphology ofspindle to elongated fibroblast-like cells (Scale bar = 2 µm). (C) Representative crystal violet staining image of PDLSCs colonies obtained by enzymatic digestion and explant methods and cultured in either FBS or PL at passage 3. (D) The number of colonies obtained by enzymatic digestion and explant outgrowth in FBS or PL at passage 1, 2, 3, 4, and 5. No significant difference was observed in colony number in any of the above culture conditions. (E) Flow cytometric analysis of PDLSCs surface markers at P3 isolated by either enzymatic or explant culture and maintained in either FBS or PL. No statistically significant difference was observed in MSC surface markers expression in any of the isolated cultures.

Enzymatic-treated and explant derived cells showed a heterogeneous morphology ranging from spindle-shaped to elongated fibroblast-like cells.  Proliferation rates were also documented and demonstrated similar potencies. PDLSCs isolated by the explant method started to expand from the attached sections on day 4, after which the cells were harvested on day 14 (Fig. 1A). The same pattern of cell growth was observed for PDLSCs isolated by enzymatic digestion method (Fig. 1B). However, the rate of cell growth was slightly higher in enzymatically-treated compared to explant derived cells, which is in line with a previously reported data (Tanaka et al., 2011).

### Colony-forming capacity of PDLSCs

Colony forming cells are considered a prerequisite for osteooblast differentiation (Couble et al., 2000; Huang et al., 2006; Papaccio et al., 2006). To assess the capacity of self-renewal of the isolated PDLSCs; a colonogenic assay was performed at passages 1 through 5. As shown in Fig. 1C, enzymatic and explant PDLSCs generated the similar number of colonies. While the use of 10% FBS showed a slightly higher number of colonies compared to 5% PL. However, the difference in colony number was not statistically significant. We have also observed that colonies grown in 10 % FBS were more compact compared to 5% PL (Fig. 1D).

### Surface markers analysis

Homogeneous cell population is required for successful use in therapeutic applications. To ensure the homogeneity of PDLSCs, the MSC markers expression profile was assessed by flow cytometry at P3. Flow cytometric analysis revealed that cells isolated by both methods and treated with either PL or FBS showed positive expression of MSC markers (CD44, CD105, CD90 and CD73) and negative for hematopoietic stem cell markers (CD45, CD34, CD11b and HLA-DR) as shown in Table 1 & Table S2 and Fig. 1E. These results indicate that the cells have similar expression profile to MSCs. No statistically significant difference was observed in any of the analyzed samples.

**Table 1:** Surface markers analysis for PDLSCs obtained by enzymatic digestion and Explant methods treated with FBS and PL.

**Extraction method**	**CD105**		**CD44**		**CD90**		**CD73**		**Negative cocktail**	
****	**PL**	**FBS**	**PL**	**FBS**	**PL**	**FBS**	**PL**	**FBS**	**PL**	**FBS**
**PDL-Enzymatic**	95.46%	91.10%	99.33%	95.90%	99.70%	98.80%	99.86%	99.57%	0.13%	0.47%
**PDL-Explant**	94.70%	95.46%	98.46%	99.33%	98.46%	99.70%	98.73%	99.87%	3%	0.13%

### Proliferative potential of PDLSCs

**Figure 2:**
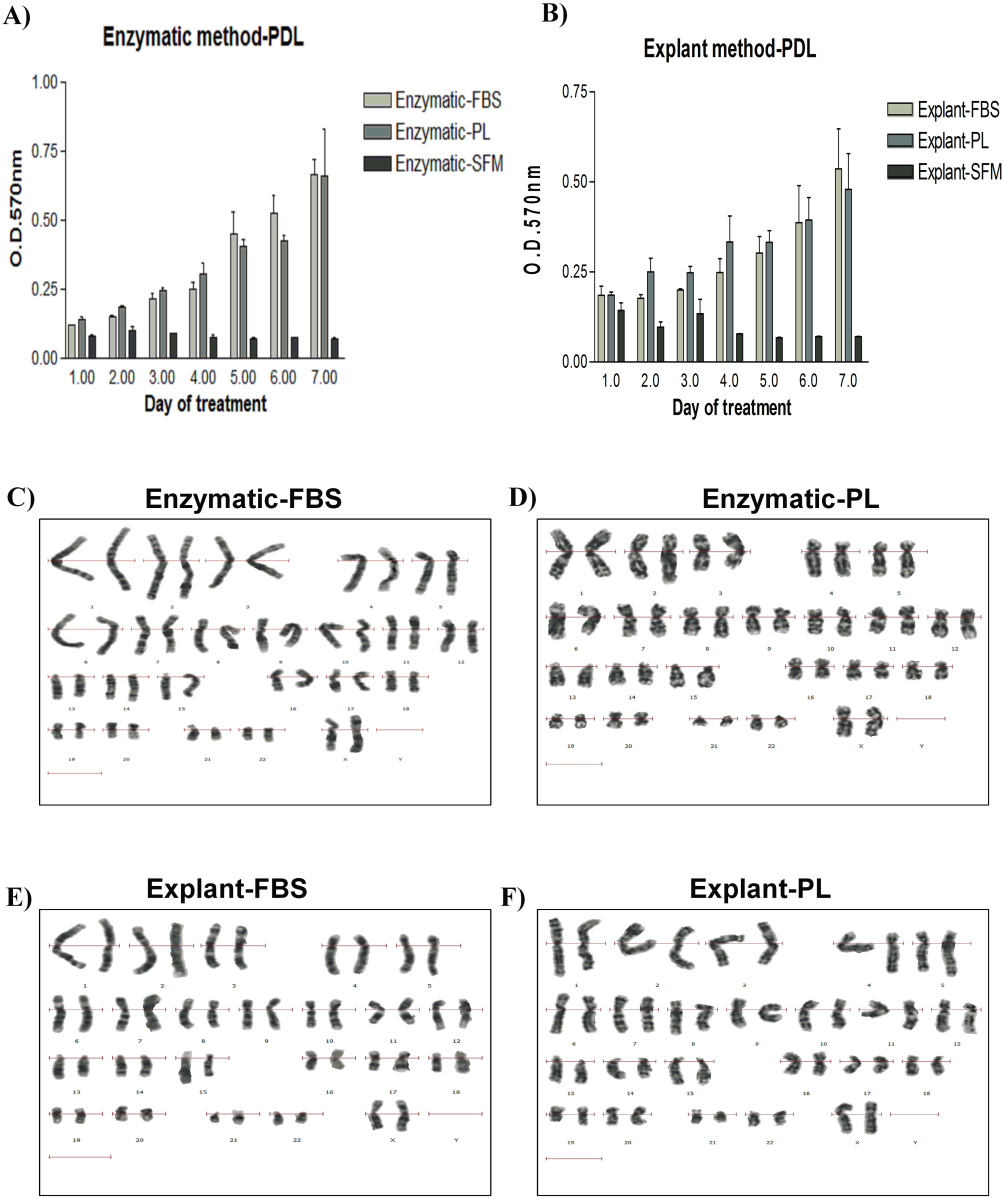
Evaluation of the proliferation potential and genetic stability of PDLSCs. Cell proliferation assay on PDLSCs at P3 isolated by either (A) enzymatic digestion or (B) explant method. Proliferation potential was measured daily for 7 days. Cells were cultured in FBS or PL and serum free medium was used as a negative control. No statistically significant difference in cell proliferation was observed under any of the culture conditions. (C & D) Karyotype analysis of PDLSCs at P7 obtained by enzymatic digestion cultured in FBS or PL respectively. (E & F) Karyotype analysis of PDLSCs at P7 obtained by explant method cultured in FBS or PL respectively. All isolated PDLSCs exhibited a normal karyotype without showing any aneuploidy, polyploidy or chromosome structural abnormality in metaphases.

To investigate the effect of the isolation method on the proliferative potential of PDLSCs, proliferation rates of the isolated PDLSCs were investigated using the CellTiter 96® Non-Radioactive Cell Proliferation Assay. Proliferation of PDLSCs isolated by enzyme-digestion method was slightly higher compared to PDLSCs obtained by the explant method (Figs. 2A & 2B). Similarly, FBS serum promoted a small increase in the proliferation rates when compared to PL in both methods. However, these differences were not statistically significant (Figs. 2A & 2B) (*p* value > 0.05) (Table S3).

### Karyotyping analysis

To ensure that the cell culture and expansion conditions have maintained genome stability of the isolated cells, we performed a cytogenetic analysis using the classical GTG (G-bands after trypsin and giemsa) banding technique. All isolated PDLSCs exhibited 46 chromosomes including sex chromosomes, without any aneuploidy, polyploidy or chromosomal abnormalities in metaphases at passage 3, 5 and 7 (Figs. 2C–2F).

**Figure 3:**
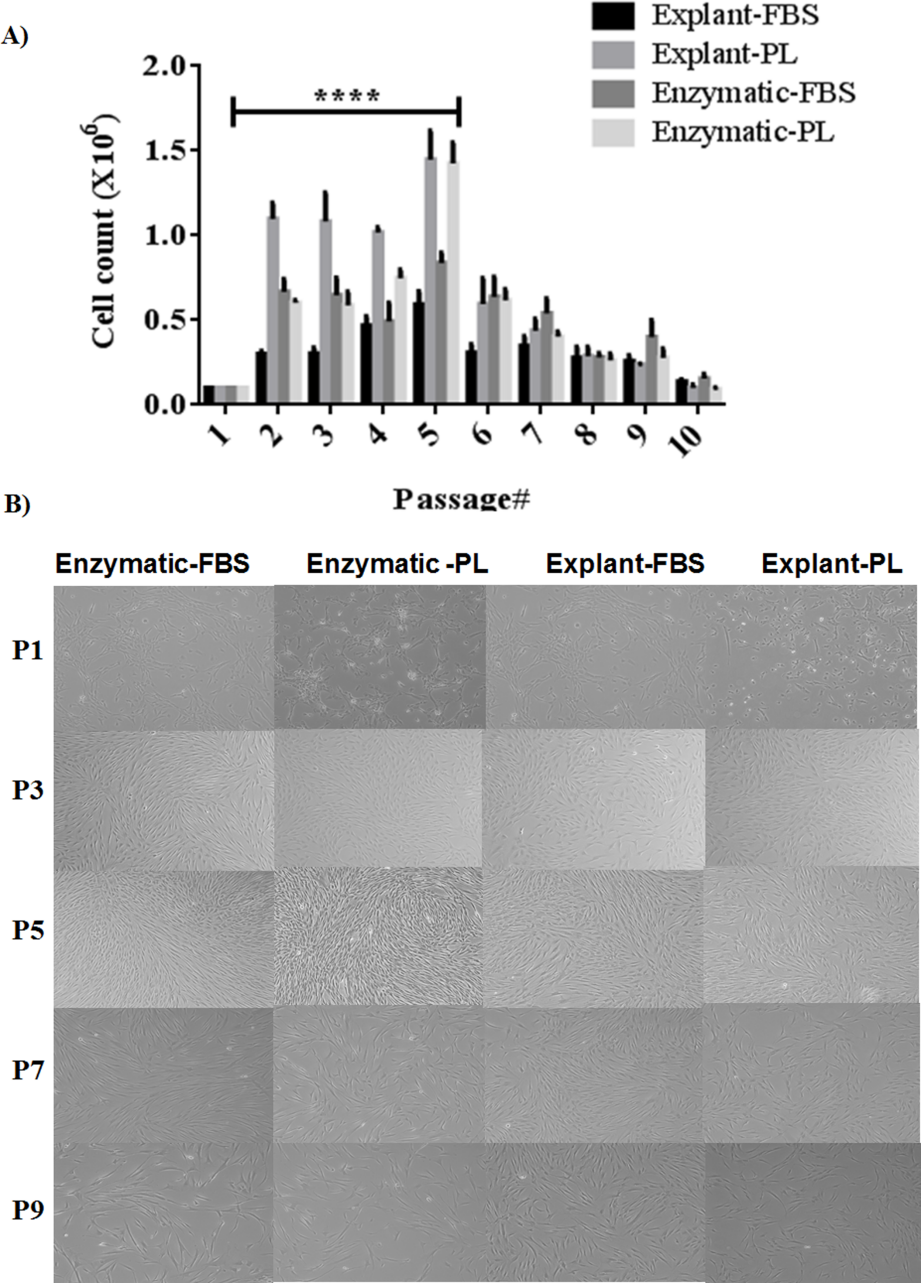
Evaluation of stemness characteristics of PDLSCs. (A) Determination of the stemness characteristics of PDLSCs isolated by two extraction methods and cultured in FBS or PL for P1–P10. PL significantly enhanced the proliferation of cells obtained by both extraction methods (*P* < 0.05) till passage 5 when compared to FBS. (B) Morphological appearance of PDLSCs obtained by two extraction methods and cultured in the presence of FBS or PL at passage P1, P3, P5, P7 and P9.

### Stemness characteristics

PDLSCs need to retain their stemness characteristics, in order to maintain them in the undifferentiated state. Stemness potential was investigated by evaluating the cell proliferation using the following assays: performing cell count, assessing MSC marker expression and observing the morphological changes for up to P10. We found that PL significantly enhanced the proliferation of the derived cells in both methods and until P5 (*p* < 0.05) (Fig. 3A) (Table S4). Moreover, our results showed that cells isolated by both methods and treated with either PL or FBS showed positive expression of MSC markers (CD44, CD90 and CD73) and negative expression for hematopoietic stem cell markers (CD45, CD34, CD11b and HLA-DR) through passage 1–10 (Table S5). This effect was more consistent in PDLSCs isolated by explant method. After P5, a gradual loss of proliferation potential was observed till passage 10 using the two methods of isolation and both serum types. Typically, CD105 expression had dropped clearly in cells derived from explant culture compared to the enzymatically derived cells (Table S5).

Morphologically, we did not observe any difference in cell shape in any of the culture conditions from passages 3 through 5 (Fig. 3B). However, cells from both cultures changed their morphology after P7 (Fig. 3B), and a loss of homogeneity was observed in culture until P10. These changes can be attributed to cell senescence or spontaneous differentiation (Gaebel et al., 2011; Kern et al., 2006) and it might be an outcome of the loss of CD105 marker.

### Osteogenic differentiation

**Figure 4:**
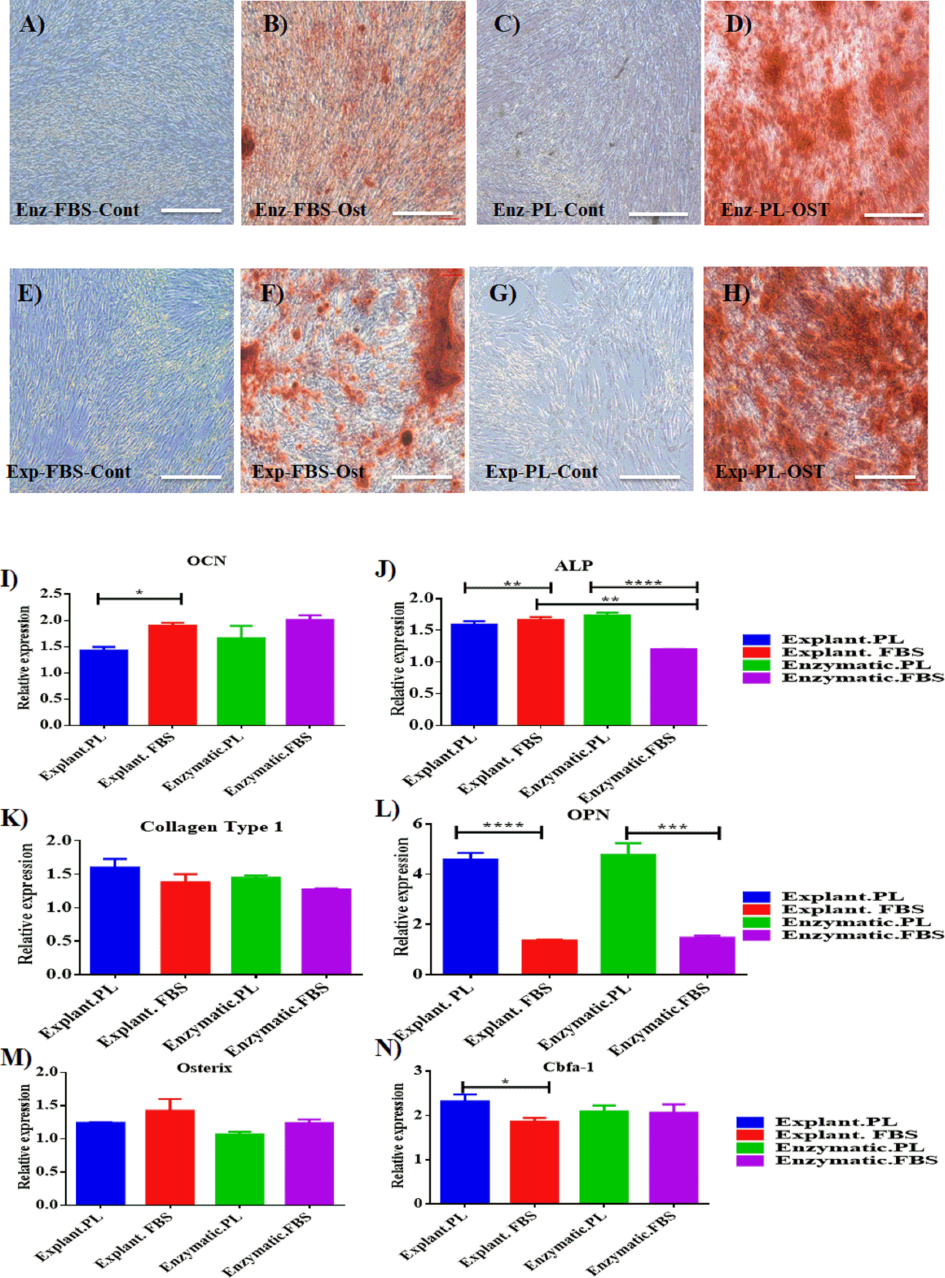
Evaluation of osteogenic differentiation potential of the isolated PDLSCs. (A, B, C, D) Osteogenic Alizarin red staining of PDLSCs cultured in osteogenic media at P3 isolated by enzymatic digestion and cultured in either FBS or PL and their control cells (cells cultured in osteogenic supplements-free media). (E, F, G, H) Alizarin red staining of PDLSCs at P3 isolated by explant outgrowth and cultured in either FBS or PL and their control cells (cells cultured in osteogenic supplements-free media). (I-N) qPCR results of osteogenic markers expression as the following: (I) OCN, (J) ALP, (K) Collagen Type 1, (L) OPN, (M) Osterix and (N) Cbfa-1, at day 14 of the osteogenic differentiation procedure. The relative expression was normalised to cDNAs of cells cultured in osteogenic supplements free media (blank control) harvested at day 14, under the same culture condition which is normalised to 1. PL significantly enhanced the expression of osteogenic markers obtained by both extraction methods compared to FBS (*P* < 0.05).

Mineralization potential of PDLSC was also assessed to evaluate their regenerative potential. Osteogenic differentiation was induced for 14 days and calcium deposits were detected with Alizarin red staining (pH 4.2; Sigma-Aldrich). Both explant and enzymatic PDLSCs exhibited an efficient osteogenic differentiation potential by showing the same intensity of alizarin red stain compared to cells cultured under the same conditions and maintained in osteogenic supplements-free media (Blank Control) (Figs. 4A–4H).

At the gene level, cells derived from any of the two extraction methods and treated with either PL or FBS showed a significant increase in the expression of the following osteogenic genes: ALP, OPN, collagen type 1, and osterix when compared to control cells derived from day 14 (*P* < 0.05) (Fig. S1A-S1E) (Table S6). Whereas, the expression of Cbfa-1 has slightly increased insignificantly in differentiated cells compared to control cells, irrespective of the extraction method and the serum type used (*P* > 0.05) (Fig. S1F).

To determine the impact of using different extraction methods and different serum types on the efficiency of osteogenic differentiation, the expression levels of the osteogenic markers of each group were normalized to their blank controls harvested on day 14 of the osteogenic differentiation. First, the expression levels were compared between differentiated cells derived by the same extraction method and treated with different serum types. For explant method, cells treated with PL showed a statistically significant increase in the expression levels of OPN and Cbfa-1 compared to FBS (*P* < 0.05), Table 2, Figs. 4L & 4N. While, OCN level was higher in cells treated with FBS compared to PL (Table 2, Fig. 4I). For enzymatic method, both ALP and OCN were significantly increased in cells treated with PL compared to FBS (Table 2, Figs. 4I & 4L). After that, we performed a comparison between cells treated with the same serum type and different extraction methods. Interestingly, for both PL and FBS cells extracted by explant method showed a statistical significant increase in the expression of ALP compared to cells derived by enzymatic method (*P* < 0.05), (Table 3, Fig. 4L), which suggests that the combination between explant extraction method and PL is more efficient on osteogenic differentiation compared to the enzymatic derived cells, and FBS treated cells.

## Discussion

Efficient expansion of the stem cells is directly related to their therapeutic potential applications in regenerative medicine (Perin, Geng & Willerson, 2003). In this study, two PDLSCs isolation methods have been employed, the explants and the enzyme digestion methods. The explant method has previously been suggested as the method of choice for efficient stem cell isolation for clinical settings, which results in an increase of cell proliferation and viability which can be lower with enzyme treatment (Huang et al., 2006). On the other hand, to reduce the risk of transmissible agents from xenogenic products, the use of PL has been previously adapted efficiently to replace the use of FBS (Abuarqoub, Awidi & Abuharfeil, 2015; Gombold et al., 2014; Tarle, Shi & Kaigler, 2011). However, a comprehensive investigation of extraction methods and the preferred serum type for cell expansion has not been studied intensively on PDLSCs. Therefore, in this study, we compared the characteristics of PDLSCs isolated by the aforementioned extraction methods and cultured in two serum types, in order to optimize cell yield and maintain stemness characteristics.

In line with other previous studies, proliferation rates and cell morphological appearance were comparable between the two extraction methods (Huang et al., 2006; Tanaka et al., 2011). We did not observe any statistically significant difference in colony forming efficiency, surface marker analysis, genomic stability and differentiation potential.

**Table 2:** Comparison of fold changes of the expression levels of osteogenic markers and *P*-value, for the differentiated cells derived from by the same extraction method and treated with two different serum types either PL or FBS (*P* < 0.05).

****	****	**OCN**		**OPN**		**ALP**		**Collagen Type1**		**Cbfa-1**		**Osterix**	
**Extraction method**	**Type of serum**	**Fold Change**	***P*- Value**	**Fold Change**	***P*- Value**	**Fold Change**	***P*- Value**	**Fold Change**	***P*- Value**	**Fold Change**	***P*- Value**	**Fold Change**	***P*- Value**
**Explant**	**PL-Ost**	1.43	*	4.58	****	1.59	ns	1.60	ns	2.32	*	1.24	ns
	**FBS-Ost**	1.90		1.35		1.67		1.38		1.86		1.42	
**Enzymatic**	**PL-Ost**	1.66	ns	4.76	****	1.74	****	1.45	ns	2.09	ns	1.07	ns
	**FBS-Ost**	2.01		1.47		1.20		1.27		2.07		1.24	

**Table 3:** Comparison of fold changes of the expression levels of osteogenic markers and *P*-value, for the differentiated cells treated with the same serum type and derived by two different extraction methods (*P* < 0.05).

****	****	**OCN**		**OPN**		**ALP**		**Collagen Type1**		**Cbfa-1**		**Osterix**	
**Type of serum**	**Extraction method**	**Fold Change**	***P*- Value**	**Fold Change**	***P*- Value**	**Fold Change**	***P*- Value**	**Fold Change**	***P*- Value**	**Fold Change**	***P*- Value**	**Fold Change**	***P*- Value**
**PL**	**Explant**	1.43	ns	4.58	ns	1.59	**	1.60	ns	2.32	ns	1.24	ns
	**Enzymatic**	1.66		4.76		1.74		1.45		2.09		1.07	
**FBS**	**Explant**	1.90	ns	1.35	ns	1.67	****	1.38	ns	1.86	ns	1.42	ns
	**Enzymatic**	2.01		1.47		1.20		1.27		2.07		1.24	

Interestingly, PL has significantly enhanced the proliferation of PDLSCs derived by the explant method in passage 3 through 5, when compared to cells cultured in FBS and extracted by the enzymatic digestion method. After passage 5 and regardless of the extraction method and serum type used, the isolated cells started to change their morphological appearance and their expression potential of MSC markers with a gradual loss of CD105 expression. Loss of CD105 expression during culture has been reported in both mouse and human MSCs, and our result is in line with these studies (Feng et al., 2010; Zhu et al., 2010). As a result, a decrease in cell proliferation either due to culture confluence, senescence induction or spontaneous differentiation could likely explain the loss of CD105 expression on PDLSCs (Gaebel et al., 2011; Kern et al., 2006). Thus, as CD105 expression is of importance for the therapeutic efficacy of MSCs, cell confluence and culture time should be carefully controlled (Anderson et al., 2013).

At the molecular level, the expression levels of osteogenic markers; OCN, ALP, OPN, Collagen type1 and osterix were significantly increased in differentiated cells compared to the undifferentiated control cells extracted by explant or enzymatic protocols, and treated with different serum types. However, Cbfa-1 did not show any statistical increase among the differentiated groups when compared to their control groups, this could be explained by the fact that Cbfa-1 expression is upregulated in preosteoblasts and downregulated in mature osteoblasts (Komori, 2019). Based on that, Cbfa-1 is very important to enhance the proliferation of osteoblast progenitors and their differentiation into mature osteoblasts by controlling the expression of other osteogenic markers (Komori, 2019).

In addition, we found a significant increase in gene expression level of OPN, ALP, and Cbfa-1 in differentiated cells isolated by any of the extraction methods and expanded in PL when compared to cells cultured in FBS. While an increase in the expression of OCN and Osterix genes was detected when cells cultured in FBS. One possible explanation for this difference is the level of cell maturation during the process of osteogenesis. It is likely that during the differentiation to osteoblasts, the isolated cells were at different stages of the differentiation process due to the differences in their proliferative capacities (Neve, Corrado & Cantatore, 2011). So, this might be related to the reduction in the proliferative ability of cells. By increasing the differentiation period, this might result in increasing the level of expression of osteogenic markers. Another explanation can be related to the cells’ microenvironment. The properties of cells depend upon the gene expression regulated by intercellular environment and intracellular signals (Comoli et al., 2008). Any change in the surrounding environment may alter the level of expression, which in turn change the cell characteristics (Comoli et al., 2008). At the cellular level, the two different culture methods provide dramatically different microenvironments. In relation to this, the enzymatic digestion method disrupts intercellular connections, whereas the explants method maintains cellular connections (Jang et al., 2016). This fundamental difference in the microenvironments might be considered as a contributing factor to the difference in osteogenic markers expression levels of PDLSCs isolated by the two protocols.

In order to reduce the risk of transmissible agents from xenogenic products, we compared the cell proliferation of two protocols cultured under FBS or PL. We found that the proliferation rate was higher in cells derived from any of the extraction protocols and using PL compared to FBS. However, cell proliferation only reached statistical significant level using the explant method. This would suggest that the combination between explant derived cells and PL can serve as an alternative to the use of cells derived enzymatically and treated with FBS.All other characteristics were comparable using any culture conditions, indicating that PL is an acceptable and even preferable alternative to FBS.

In conclusion, PDLSCs extracted by any of the two extraction methods can be maintained in xeno-free product without affecting their proliferation potential and stemness characteristics. In this case, PL can be used as an alternative source to FBS for propagation of PDLSCs. In addition, based on all of the evaluation assays and differentiation potential, the cells isolated by explant method and propagated in PL can be efficiently used to generate osteoblasts. Thus, we propose that this combination serves as an efficient binomial strategy, that can be used to promote bone growth and repair in the near future of regenerative medicine.

##  Supplemental Information

10.7717/peerj.7465/supp-1 Figure S1 Evaluation of osteogenic differentiation potential of the isolated PDLSCs compared to thier control cells qPCR results of osteogenic markers expression as the following: (A) OCN, (B) ALP, (C) Collagen Type 1, (D) OPN, (E) Osterix and F) Cbfa-1, at day 14 of the osteogenic differentiation procedure. The relative expression levels of these genes were normalized to cDNA samples of cells cultured under the same conditions, harvested at day 1 of the osteogenic differentiation process.

10.7717/peerj.7465/supp-2 Table S1 Primers of osteogenic differentiation markers

10.7717/peerj.7465/supp-3 Table S2 Surface markers analysis Raw data of surface markers analysis of PDLSCs extracted by two different methods and treated with different serum types at P3.

10.7717/peerj.7465/supp-4 Table S3 Cell proliferation assay (MTT) Raw data of cell proliferation assay of PDLSCs extracted by two different methods and treated with different serum types at P3.

10.7717/peerj.7465/supp-5 Table S4 Stemness assay results of PDLSCs isolated by two different extraction methods and cultured in two different serum types for Passage 1 until Passage 10

10.7717/peerj.7465/supp-6 Table S5 Surface markers analysis at different passages Surface markers analysis for PDLSCs obtained by enzymatic digestion and outgrowth methods treated with FBS and PL at different passages.

10.7717/peerj.7465/supp-7 Table S6 Expression levels of osteogenic markers Raw data of the expression levels of osteogenic markers expressed by the isolated PDLSCs after 14 days of osteogenic differentiation compared to their Blank control.

## Additional Information and Declarations

### Data Availability

The following information was supplied regarding data availability: Raw data are available as Supplemental Materials.
