# Supplementary figures and images for: The effect of platelet lysate in culture of PDLSCs: an in vitro comparative study
Source: PeerJ. 2019 Aug 8;7:e7465. doi: 10.7717/peerj.7465 (PMC6689390; doi:10.7717/peerj.7465)

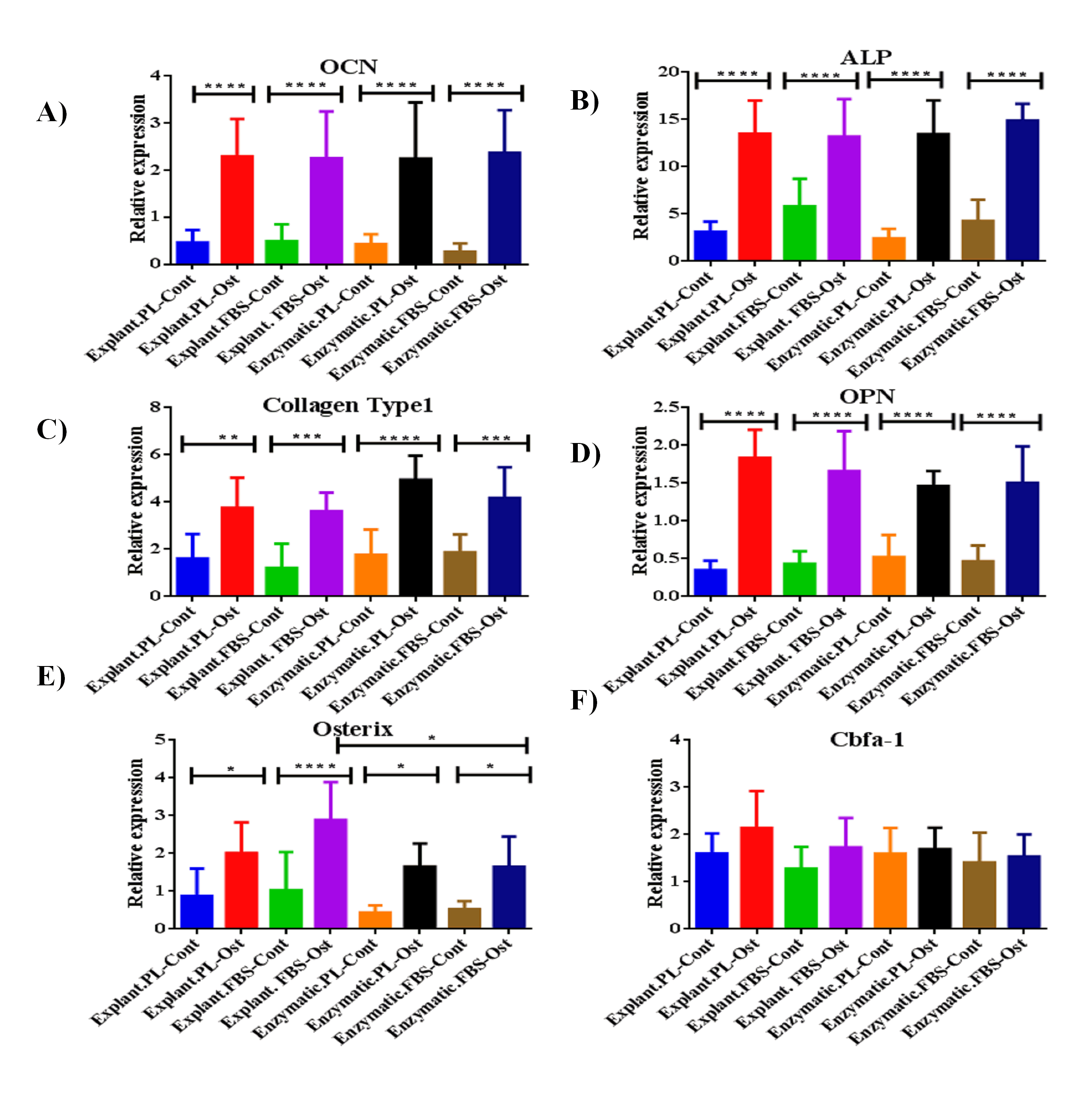

Supplement: Figure S1 — qPCR results of osteogenic markers expression as the following: (A) OCN, (B) ALP, (C) Collagen Type 1, (D) OPN, (E) Osterix and F) Cbfa-1, at day 14 of the osteogenic differentiation procedure. The relative expression levels of these genes were normalized to cDNA samples of cells cultured under the same conditions, harvested at day 1 of the osteogenic differentiation process. [file peerj-07-7465-s001.png]
