# Supplementary material for: The effect of platelet lysate in culture of PDLSCs: an in vitro comparative study
Source: PeerJ. 2019 Aug 8;7:e7465. doi: 10.7717/peerj.7465 (PMC6689390; doi:10.7717/peerj.7465)
Supplement: Table S1 [file peerj-07-7465-s002.docx]

**Table 1**: Primers of osteogenic markers.

| **Gene** | **Forward** | **Reverse** |
| --- | --- | --- |
| **ALP** | AGTAGGGCCTGGATC TTC TT | CTGCTTCTCAGTCAG AAGGT |
| **OPN** | TGCAGCCTTCTCAGCCAA | GGAGGCAAAAGCAAATCACTG |
| **OCN** | GACGAGTTGGCTGACCACA | CAAGGGGAAGAGGAAAGAAGG |
| **Cbfa-1** | ATGTGTGTTTGTTTCAGCAGCA | TCCCTAAAGTCACTCGGTATGTGTA |
| **Collagen type 1** | GGAGATGATGGGGAAGCTGG | TTGGCACCATCCAAACCACT |
| **Osterix** | TAATGGGCTCCTTTC ACCTG | CACTGGGCAGACAGT CAGAA |
| **PPIA cyclophillin** | TCCTGGCATCTTGTCCATG | CCATCCAACCACTCAGTCTTG |
