# Supplementary material for: The effect of platelet lysate in culture of PDLSCs: an in vitro comparative study
Source: PeerJ. 2019 Aug 8;7:e7465. doi: 10.7717/peerj.7465 (PMC6689390; doi:10.7717/peerj.7465)
Supplement: Table S5 — Surface markers analysis for PDLSCs obtained by enzymatic digestion and outgrowth methods treated with FBS and PL at different passages. [file peerj-07-7465-s006.docx]

| **Negative cocktail** | | **CD73** | | | **CD90** | | **CD44** | | | **CD105** | | | **Extraction method** | **Passage #** |
| --- | --- | --- | --- | --- | --- | --- | --- | --- | --- | --- | --- | --- | --- | --- |
| **FBS** | **PL** | | **FBS** | **PL** | **FBS** | **PL** | | **FBS** | **PL** | | **FBS** | **PL** |  |  |
| 2% | 2% | | 100% | 100% | 99% | 100% | | 99% | 100% | | 75.6% | 76% | **PDL-Enzymatic** | **P1** |
| 2% | 6% | | 98% | 98% | 98.3% | 100% | | 99.3% | 99% | | 60% | 75% | **PDL-Explant** |  |
| 0.47% | 0.13% | | 99.57% | 99.86% | 98.80% | 99.70% | | 95.90% | 99.33% | | 91.10% | 95.46% | **PDL-Enzymatic** | **P3** |
| 0.13% | 3% | | 99.87% | 98.73% | 99.70% | 98.46% | | 99.33% | 98.46% | | 95.46% | 94.70% | **PDL-Explant** |  |
| 0.3% | 0.1% | | 87.6% | 90.6% | 100% | 100% | | 99.9% | 99.9% | | 28.2% | 28.1% | **PDL-Enzymatic** | **P5** |
| 0.1% | 0% | | 99.8% | 99.0% | 99.9% | 99.3% | | 98.8% | 91.8% | | 27.3% | 36.9% | **PDL-Explant** |  |
| 0.1% | 0.2% | | 100% | 93.2% | 100% | 99.9% | | 99.9% | 99.77% | | 71.9% | 83.2% | **PDL-Enzymatic** | **P7** |
| 0.1% | 0.1% | | 94.9% | 91.9% | 99.9% | 99.9% | | 99.4% | 98.6% | | 67.3% | 37.2% | **PDL-Explant** |  |
| 0% | 1% | | 71.5% | 53.6% | 100% | 99.9% | | 99.9% | 99.8% | | 76.6% | 67.7% | **PDL-Enzymatic** | **P9** |
| 0.6% | 0.1% | | 98.3% | 100% | 97.7% | 100% | | 92.2% | 97% | | 33.3% | 27.7% | **PDL-Explant** |  |

**Table 3:** Surface markers analysis for PDLSCs obtained by enzymatic digestion and outgrowth methods treated with FBS and PL at different passages.
